# Transcranial direct current stimulation for enhancing attention following mild traumatic brain injury: a narrative review

**DOI:** 10.3389/fneur.2026.1792457

**Published:** 2026-04-30

**Authors:** Henry Abumohor, Stephen De Souza

**Affiliations:** 1 Faculty of Medicine, Medical Research Club, Al-Quds University, Jerusalem, Palestine; 2 Somerset NHS Foundation Trust, Taunton, United Kingdom

**Keywords:** attention deficits, cognitive function, mild traumatic brain injury, neurocognitive outcome, persistent post-concussive symptoms, transcranial direct current stimulation

## Abstract

**Background:**

Attention deficits often persist after mild traumatic brain injury (mTBI), limiting recovery despite normal imaging. Transcranial direct current stimulation (tDCS) has emerged as a non-invasive therapeutic option.

**Aims:**

This review evaluates the evidence supporting tDCS as a method to improve attention in individuals with mTBI or post-concussive symptoms.

**Methods:**

Peer-reviewed studies focusing on attentional outcomes after tDCS in mTBI were examined. Related findings from ADHD and mild cognitive impairment were included where relevant.

**Results:**

Most protocols used anodal tDCS targeting the left dorsolateral prefrontal cortex (2 mA for ~20 min across multiple sessions). Reported benefits included improved sustained attention, quicker response times, and enhanced cognitive control, particularly when paired with cognitive exercises. Neurophysiological markers often reflected behavioral gains, though most trials were small and lacked follow-up.

**Conclusion:**

While preliminary results are encouraging, larger and standardized trials are essential before routine clinical use.

## Introduction

Concussions, or mild traumatic brain injuries (mTBI), represent one of the most common neurological conditions worldwide, with an estimated 69 million cases annually. Although most individuals recover within days to weeks, a significant proportion—approximately 15–20%—experience persistent cognitive and neuropsychiatric symptoms well beyond the acute phase (1). Among these, attentional deficits are particularly prominent and can substantially interfere with academic performance, workplace productivity, and daily functioning (2).

Attention is a multidimensional construct encompassing sustained attention (maintaining focus over time), selective attention (filtering distractions), divided attention (managing multiple tasks simultaneously), and attentional switching (shifting between mental sets). These domains are typically assessed using validated neuropsychological measures such as the Continuous Performance Test (CPT), which evaluates sustained vigilance; the Trail Making Test, which probes attentional switching and cognitive flexibility; and the Attention Network Test (ANT), which distinguishes between alerting, orienting, and executive control components of attention. Linking these measures to theoretical subdomains provides a framework for understanding the specific attentional impairments observed after mTBI (3–5).

Despite the clinical significance of attentional dysfunction, conventional neuroimaging often fails to reveal structural abnormalities in patients with lingering symptoms. Advanced imaging techniques, however, suggest that mTBI disrupts white matter integrity and functional connectivity within the frontoparietal attention network, particularly involving the dorsolateral prefrontal cortex (DLPFC). These network-level disruptions provide a mechanistic explanation for persistent attentional difficulties and highlight the need for interventions that directly target cortical excitability and connectivity (4, 6).

In the context of mTBI, even mild biomechanical forces can disrupt white matter tracts and impair the functional connectivity among these regions, especially within the frontoparietal attention network. These disruptions often occur at a microstructural level, which means that they may not be detectable with standard neuroimaging but still lead to clinically significant attentional impairments (6). This disconnect between structural findings and cognitive symptoms underscores the value of performance-based assessments and supports targeting attention networks directly—through interventions such as cognitive training and neuromodulation (7, 8).

Transcranial direct current stimulation (tDCS) has emerged as a promising non-invasive neuromodulation technique capable of modulating cortical excitability and supporting cognitive recovery. Preliminary studies suggest that anodal stimulation of the left DLPFC may enhance sustained attention, reaction time, and task-switching performance, particularly when paired with cognitive training. tDCS is particularly suited to modulating distributed attentional networks, as it delivers low-intensity currents across broader cortical regions, unlike focal techniques such as TMS or tACS. However, most existing reviews have focused broadly on cognitive outcomes after mTBI rather than isolating attention as a distinct domain (9, 10).

Attentional dysfunction following mTBI shares neurobiological and functional similarities with other conditions characterized by prefrontal network disruption, such as Attention-Deficit/Hyperactivity Disorder and Mild Cognitive Impairment. In particular, these conditions involve overlapping alterations in frontoparietal and dorsolateral prefrontal cortex (DLPFC) networks that underlie executive and attentional control, providing a rationale for drawing on evidence from these populations to inform tDCS-related mechanisms and outcomes (6, 8).

Given these gaps, the present narrative review aims to critically evaluate the role of transcranial direct current stimulation in enhancing attentional function in individuals with mild traumatic brain injury or persistent post-concussive symptoms. Specifically, the review synthesizes evidence on whether tDCS, compared to sham stimulation or alternative cortical targets, improves attentional domains such as sustained, selective, divided, and switching attention. By integrating findings across clinical populations and stimulation protocols, we clarify the potential of tDCS as a targeted intervention for attentional recovery and identify directions for future research (9).

## Methods

This narrative review was conducted to synthesize and critically examine the current evidence regarding the use of tDCS for improving attentional function in individuals with mTBI or concussion. A comprehensive literature search was performed across four major electronic databases: PubMed, Web of Science, ScienceDirect, and Google Scholar. Search terms were constructed using combinations of Medical Subject Headings and keywords, including “transcranial direct current stimulation” OR “tDCS,” AND “mild traumatic brain injury” OR “concussion,” AND “attention,” “cognitive function,” or “neurocognitive outcome.” Boolean operators were employed to refine and optimize search results. The search was conducted between August 2025 and September 2025 and finalized on September 30, 2025. A total of 50 records were retrieved initially, of which 25 were excluded after title and abstract screening, leaving 25 full-text articles that met the inclusion criteria and were synthesized in the present review.

Filters were applied to restrict the selection to human studies published in English between 2010 and 2025. The decision to restrict the search to studies published from 2010 onward was based on methodological advances in tDCS research during this period. Since 2010, stimulation protocols have become more standardized (e.g., electrode montages, sham controls), and clinical applications in mTBI have expanded significantly. Earlier studies often lacked these methodological refinements, making them less comparable to contemporary practice. Therefore, limiting the search to 2010–2025 ensured that the included evidence reflects current approaches and clinically relevant findings. To ensure thoroughness, the reference lists of relevant articles and reviews were also manually screened for additional studies.

Studies were included if they were original research articles utilizing randomized controlled trials, cohort studies, case–control designs, or pre–post interventions evaluating the effects of tDCS in individuals with mTBI or concussion. Eligible studies were required to report attention as a primary or secondary outcome and be published in peer-reviewed journals with full-text availability. For the purposes of this review, attention outcomes were defined as performance on validated neuropsychological tasks such as CPT for sustained vigilance, Trail Making Test for attentional switching, Stroop task for selective attention, and ANT for alerting and executive control, as well as objective behavioral measures including reaction time and error rates, or structured symptom scales specifically assessing attentional complaints. Studies reporting only global cognitive outcomes without any attention-related or closely associated cognitive measures (e.g., working memory, processing speed, or executive control) were excluded. In addition to studies involving mTBI populations, evidence from related clinical populations, including Attention-Deficit Hyperactivity Disorder and Mild Cognitive Impairment, was considered to provide mechanistic and contextual support, particularly in areas where mTBI-specific evidence remains limited. Selection of these contextual studies was based on relevance to attentional networks, similarity of neurocognitive outcomes, and use of comparable tDCS protocols. This cross-diagnostic approach was adopted due to shared involvement of prefrontal attentional networks and the relative scarcity of directly comparable mTBI-specific tDCS studies. Exclusion criteria encompassed editorials, commentaries, animal studies, and investigations that did not report attention-specific outcomes.

To address the limited availability of strictly attention-focused studies in mTBI, the included literature spans a spectrum of evidence. Studies were interpreted and synthesized across three conceptual categories: direct evaluations of attention-related outcomes in mTBI or post-concussive populations, investigations in broader TBI cohorts or those assessing related cognitive domains closely linked to attention (such as working memory, processing speed, or executive control), and indirect or mechanistic evidence derived from other clinical populations or neuromodulation approaches. These categories were not applied as strict inclusion strata but rather served as an interpretive framework to contextualize findings. Accordingly, results are discussed with attention to the degree of directness to the primary research question.

Study selection followed a two-phase process. Initially, titles and abstracts of all retrieved records were screened by the lead reviewer for relevance. Full texts of potentially eligible studies were then assessed based on predefined inclusion and exclusion criteria. Where there were questions about the eligibility of a study, this was reviewed by a second author.

Due to heterogeneity in study methodologies, stimulation parameters, and outcome measures, a meta-analysis was not feasible. Instead, a qualitative narrative synthesis was conducted to evaluate trends and patterns across studies. Results were categorized based on reported improvements in attentional performance, variations in stimulation approaches, consistency of findings across different populations and study designs, and integration of neurophysiological markers when available. Although formal bias assessment tools were not applied given the narrative nature of this review, methodological transparency was maintained through clear reporting of selection processes and extracted data. The limitations inherent to narrative reviews such as the potential for publication bias and the absence of pooled effect sizes are acknowledged.

## Results

To provide a structured synthesis of the available evidence, the key characteristics of primary studies evaluating transcranial direct current stimulation (tDCS) or related neuromodulation techniques for cognitive or attentional outcomes are summarized in Table 1.

**Table 1:** Studies evaluating tDCS and Related neuromodulation for attention and related cognitive outcomes in mTBI and related populations.

Author (Year)	*N*	Population/Injury phase	Montage/Target	Intensity	Total sessions	Task pairing	Primary cognitive/Attentional outcome	Direction of effect
Liu et al., 2025 (2)	20	Adults with a concussion	HD-tDCS over the motor cortex	2 mA	1	Postural control tasks	Cognitive-motor integration and dynamic postural control	Improved vs. sham
Quinn de Launay et al., 2022 (13)	19	Youth with persistent post-concussive symptoms	Left DLPFC (F3)	2 mA	5	Cognitive tasks	Working memory accuracy and reaction time	Improved RT and accuracy
Trofimov et al., 2022 (14)	15	Adults with mild TBI	Left DLPFC	2 mA	Multiple	None reported	Executive task performance	Improved performance
Feiz et al., 2020 (21)	40	Participants undergoing cognitive training	Left DLPFC	1–2 mA	8	Cup-stacking cognitive training	Working memory and processing speed	Moderate improvement
Qi et al., 2025 (22)	36	Elite athletes with fatigue	Dual-site tDCS (M1 or DLPFC)	2 mA	1	Stroop task	Selective attention and decision making	Improved Stroop accuracy
Sacco et al., 2016 (23)	32	Moderate–severe TBI rehabilitation patients	Left DLPFC	2 mA	10	Divided attention training	Reaction time and attentional errors	Improved attention

### Enhancing sustained attention and response speed

Across several studies, tDCS has shown potential to improve sustained attention and reaction time in individuals with mTBI or persistent post-concussive symptoms (PPCS) (11). Most studies applied anodal tDCS over the left DLPFC, generally at intensities of 2 mA, using sponge electrodes for 20-min sessions. The emerging consensus is that single-session effects are limited and short-lived, while multi-session protocols (typically 5–10 sessions) appear to yield more reliable gains in attention and cognitive control (12).

In a pediatric population with PPCS, repeated tDCS sessions improved working memory accuracy and significantly reduced reaction time, suggesting that attentional efficiency may be improved even when attention is not the primary target (13). Similarly, a trial in adults using functional near-infrared spectroscopy (fNIRS) found that repeated stimulation not only improved executive task performance but also increased prefrontal oxygenation, suggesting that attention-related brain circuits may be modulated both functionally and hemodynamically by tDCS (14).

Some studies combined tDCS with targeted cognitive training, yielding even stronger results. When stimulation was paired with attention or working memory tasks, gains were observed in complex attention domains like sustained focus and interference control. Neuroimaging data from EEG and functional magnetic resonance imaging (fMRI) in these studies further showed changes in frontoparietal network activity, pointing to enhanced functional integration across attentional systems (14–16).

Comparative evidence from older adults with mild cognitive impairment (MCI) further supports these findings. In this group, tDCS applied over the DLPFC led to better Stroop performance, increased working memory span, and improved performance on the Digit Span and Trail Making Test—all of which tap into attentional processes. Although MCI differs in etiology from mTBI, the similarity in affected networks lends support to the generalizability of tDCS effects on attentional functions (17).

### Targeting executive and cognitive control systems

While relatively few studies isolate attention as a primary outcome, many report improvements in executive function domains that heavily involve attentional control (12). For example, a randomized crossover study protocol proposed comparing stimulation over the left DLPFC and the left temporal cortex. Although the primary focus was memory, both sites are implicated in attentional functioning. As this study represents a protocol, no outcome data are currently available; however, the design reflects growing interest in evaluating whether different cortical targets may differentially influence higher-order cognitive functions (18).

Reviews that include both tDCS and Rapid Transcranial Magnetic Stimulation interventions report associations with broad cognitive gains following prefrontal stimulation, especially in populations with persistent symptoms following mTBI. While attention was not always measured separately, improvements in working memory, inhibition, and task-switching suggest that attentional networks may benefit as a secondary outcome of executive-focused stimulation (12, 16).

Other modalities like low-intensity pulsed electrical stimulation (LIP-tES) have also demonstrated potential. For instance, IASIS Neurofeedback, a form of LIP-tES combined with EEG monitoring, has been reported to reduce post-concussive symptoms and abnormal slow-wave activity. Although attention was not directly assessed, the results support the idea that neuromodulation can broadly improve cognitive functioning through mechanisms affecting attentional readiness (19).

Beyond mTBI, high-definition tDCS studies in ADHD and MCI populations have demonstrated improved attention span and reduced reaction time variability. Though orienting and alerting attention effects remain mixed, these studies underscore the capacity of tDCS to impact the executive control of attention under the right conditions (12, 15).

### The power of repetition: why session frequency matters

One of the most consistent findings across the literature is that the effectiveness of tDCS in mTBI appears to be strongly influenced by how often and how consistently stimulation is delivered. While early studies experimented with single-session protocols, these typically resulted in only mild or short-lived effects on cognitive outcomes such as reaction time and sustained attention. In contrast, protocols involving multiple sessions—ranging from five to 10 applications—are far more likely to yield meaningful and enduring improvements (12–14).

For example, studies applying 10 sessions of 2 mA anodal tDCS over the left DLPFC—whether as standalone treatment or paired with cognitive training—have consistently shown improvements in sustained attention and executive function, along with measurable neurophysiological changes such as increased prefrontal oxygenation or altered EEG patterns (14, 16). These outcomes not only support the efficacy of tDCS but also suggest cumulative neuroplastic effects over time.

Support for this time-dependent model comes from youth-focused studies as well. In adolescents with PPCS, repeated stimulation led to notable improvements in working memory and attention, outcomes that were not seen in sham groups, further reinforcing that cognitive recovery may depend on sustained intervention rather than a one-off stimulation approach. Even in more naturalistic rehabilitation settings, repeated-session protocols have proven both feasible and beneficial. A three-week intervention using 2 mA anodal tDCS three times per week showed that patients with subacute TBI not only tolerated the treatment well, but also demonstrated promising cognitive gains, especially in attention-related domains. Importantly, the neurobiological environment of subacute mTBI (weeks after injury) differs substantially from that of chronic mTBI (months to years post-injury), particularly in terms of metabolic recovery, neuroinflammation, and network reorganization. These differences may influence responsiveness to neuromodulation interventions such as tDCS (13).

Another trial combined daily stimulation with computerized training over a two-week period, targeting either the bilateral temporal cortex or left DLPFC. Although the primary endpoint was episodic memory, follow-up EEG showed enhancements in cortical rhythms (e.g., increased alpha power) and cognitive function that persisted at 3 months. These results suggest that frequent stimulation not only improves immediate performance but may also consolidate long-term neural efficiency, with possible spillover benefits for attention (20, 21).

### Location matters: targeting attention-related brain regions

Equally important to how often stimulation is delivered is where it’s delivered. The left DLPFC is by far the most common target in attention-focused tDCS research. In many experimental protocols, this region is targeted using 10–20 EEG system coordinates, typically positioning the anode at F3 with the cathode over the contralateral supraorbital region Fp2 or Fp1, and for good reason. This region is central to the regulation of attention, executive function, and working memory—domains frequently impaired after mTBI. Numerous studies and reviews consistently show that anodal stimulation of the DLPFC is associated with improved cognitive outcomes, particularly in tasks requiring sustained focus, task switching, or divided attention (12).

In one crossover study protocol, researchers proposed comparing stimulation over the DLPFC versus the left temporal cortex. Although both regions are implicated in cognitive control, the aim was to determine whether site-specific differences would yield unique performance benefits. As this study represents a protocol, no outcome data are currently available; however, it reflects interest in whether montage selection may meaningfully influence cognitive outcomes, even though attention was not the primary endpoint (18).

Innovative approaches have also explored dual-site stimulation, with some promising results. In a study involving elite athletes under fatigue, stimulation of the primary motor cortex (M1) improved selective attention performance—measured via Stroop task accuracy—more than DLPFC stimulation or sham. This may reflect motor-attentional integration, particularly under cognitively demanding conditions, and opens a new avenue for optimizing tDCS protocols in mTBI and other high-performance settings (22).

While the DLPFC remains the most commonly studied stimulation target, these emerging studies point to the importance of tailoring stimulation sites based on symptom profile and functional goals—whether executive overload, sensorimotor fatigue, or impaired attentional control (15).

### Cognitive engagement: the case for pairing tDCS with training

Beyond stimulation parameters, there is growing consensus that tDCS is more effective when paired with cognitive training. This pairing leverages Hebbian principles “cells that fire together, wire together” by activating neural networks during the window of heightened excitability induced by stimulation (16, 20).

In a double-blind trial on individuals with moderate-to-severe TBI, stimulation over the DLPFC was followed by 40 min of divided attention training. The combination yielded not only faster reaction times but also reduced errors and fewer attentional lapses. Importantly, fMRI showed that brain regions previously showing hyperactivation returned to more efficient levels of functioning, indicating network-level normalization (23).

Another study involving tDCS over the lesion-side DLPFC paired stimulation with computerized attention exercises. Participants receiving active stimulation made fewer errors and responded more quickly, while post-treatment imaging revealed reduced frontal hyperactivation. This suggests that stimulation may “prime” the cortex to better respond to structured task demands (17).

Review papers across populations—including ADHD and MCI—echo these findings. They recommend integrating cognitive or behavioral tasks that engage the same networks being stimulated, noting that doing so appears to increase both the magnitude and the durability of cognitive improvements (12, 21).

Taken together, this growing body of evidence supports the concept that tDCS is not a stand-alone intervention, but rather a neuromodulatory enhancer—one that reaches its full potential when embedded in well-timed, task-relevant rehabilitation contexts (12).

## Discussion

### Epidemiology and functional consequences of attentional impairment

Concussions are among the most frequently reported neurological events globally, with an estimated 69 million cases annually (7). Although most individuals recover within days to weeks, approximately 15–20% continue to experience persistent cognitive and neuropsychiatric symptoms well beyond the acute phase (24). One of the most prominent long-term symptoms is impaired attention, a domain critical for navigating everyday tasks, from school and work responsibilities to driving and social interactions. These deficits can present as lapses in focus, difficulty with task switching, mental fatigue, and slower response times under cognitive load—creating considerable barriers to functional independence.

Longitudinal research in both pediatric and adult populations demonstrates that attentional deficits can outlast the resolution of symptoms. For instance, even when individuals are deemed “recovered” based on symptom checklists, many continue to report cognitive fog, poor concentration, and inconsistent mental stamina (24). These lingering effects can have real-life implications such as academic failure, reduced workplace productivity, interpersonal strain, and lowered quality of life. Moreover, these issues can initiate a cascade of secondary psychosocial consequences, including anxiety, reduced self-confidence, and avoidance behaviors. As such, there is a strong clinical rationale for specifically targeting attention in both research and rehabilitation planning for individuals with mTBI (24).

### Neurobiological underpinnings: microstructure and network integrity

One of the most complex features of mTBI is the disconnect between patient-reported symptoms and normal findings on standard neuroimaging techniques such as CT or conventional MRI. However, advanced imaging modalities—particularly diffusion tensor imaging have revealed that even mild biomechanical forces can disrupt the brain’s white matter microstructure. These disruptions are especially pronounced in the superior longitudinal fasciculus, a major tract that connects the frontal and parietal lobes and supports the frontoparietal attention network (6).

Functional imaging studies corroborate these structural findings. Resting-state fMRI analyses show persistent hypo-connectivity between the thalamus and DLPFC—both crucial hubs of attentional processing—well after clinical symptoms have subsided (8). These disconnections correlate with deficits in sustained and divided attention as observed on neuropsychological tests, including the Attention Network Test and Continuous Performance Test. The implication is that attentional difficulties in mTBI arise not merely from focal lesions but from network-level disruptions in signal integration and synchronization across brain systems. This “functional disconnectivity” model provides a mechanistic explanation for why many individuals experience cognitive dysfunction in the absence of clear structural damage and highlights the potential of network-level interventions—such as neuromodulation—to restore connectivity and function.

The rationale for targeting the left dorsolateral prefrontal cortex is further supported by foundational neuroanatomy. The frontoparietal attention network integrates executive processes in the frontal cortex with orienting functions in the parietal cortex, providing the basis for sustained and selective attention. Connectivity between these regions is mediated by the superior longitudinal fasciculus, a major white matter tract linking frontal and parietal areas. Together, these systems underpin attentional regulation and provide the neurobiological basis for modulating attentional control via prefrontal stimulation, reinforcing the choice of the left DLPFC as a stimulation site (9, 12).

### Role of the DLPFC in attention and rationale for targeted stimulation

The DLPFC plays a central role in orchestrating higher-order attentional processes, including goal maintenance, response inhibition, and working memory. It serves as a cortical “control center” that interfaces with parietal regions, the anterior cingulate cortex, the thalamus, and subcortical systems like the basal ganglia to coordinate focus and suppress irrelevant stimuli (8). Within this attentional architecture, the left DLPFC is particularly involved in sustaining attention over time and managing interference—a pattern consistent across both lesion studies and functional neuroimaging.

Because of this central role, the DLPFC is the most frequently targeted region in cognitive tDCS protocols. Anodal stimulation of the left DLPFC—most commonly implemented using the International 10–20 EEG system with the anode positioned at F3 and the cathode placed over a contralateral supraorbital location such as Fp2—has been associated with increased cortical excitability and improved cognitive performance, particularly in tasks requiring attentional engagement and executive control (8). The rationale is neurophysiologically grounded: tDCS induces polarization of cortical neurons, enhancing synaptic strength via NMDA receptor activity and promoting long-term potentiation—a cellular basis of learning and plasticity (14). This makes DLPFC stimulation a promising method for reversing network inefficiencies in mTBI-related attentional impairment.

### Mechanism of action, safety, and neurobiological evidence

Current understanding of the mechanisms and safety of transcranial direct current stimulation is largely derived from studies in broader neurological and healthy populations, as mTBI-specific evidence remains limited. Anodal tDCS exerts its effects through low-intensity direct current, typically 1–2 mA, applied via scalp electrodes for approximately 15–30 min per session. Importantly, stimulation intensity is more appropriately described in terms of current density (mA/cm^2^), which depends on both the applied current and electrode surface area. In many conventional tDCS protocols using sponge electrodes of approximately 25–35 cm^2^, a 2 mA current corresponds to a current density of roughly 0.057–0.08 mA/cm^2^, although exact values vary across studies depending on montage and electrode size. This subthreshold stimulation does not directly elicit action potentials but rather modulates membrane potentials, increasing neuronal responsiveness. EEG studies have demonstrated post-stimulation increases in frontal alpha power—an oscillatory marker linked to enhanced attentional readiness (25). Meanwhile (fNIRS) has shown increases in prefrontal oxygenation following tDCS, suggesting improved neurovascular coupling and local metabolic activity (14).

Although anodal stimulation is commonly described as excitatory and cathodal stimulation as inhibitory, these effects are not universally consistent. Experimental and clinical studies have demonstrated that stimulation outcomes depend on multiple factors, including baseline cortical activity, stimulation intensity, and duration. In certain contexts, cathodal stimulation may be therapeutically beneficial by down-regulating maladaptive cortical hyperexcitability, a phenomenon reported in some cases of mild traumatic brain injury. Additionally, non-linear or “ceiling” effects have been described, whereby higher stimulation intensities or longer durations may produce paradoxical inhibitory responses rather than excitatory ones. These considerations highlight the importance of carefully optimizing stimulation parameters in clinical neuromodulation protocols (11, 20).

In terms of safety, tDCS continues to demonstrate an excellent profile. Side effects are typically limited to minor itching, tingling, or warmth at the stimulation site, with no evidence of structural damage or long-term complications even after multiple sessions (26).

### The value of repetition: multi-session protocols yield more durable gains

While early investigations into tDCS effects often relied on single-session protocols, the consensus emerging across clinical studies is clear: one-time stimulation provides little more than a fleeting benefit. Instead, meaningful and sustained cognitive improvements—particularly in attention—tend to arise only after multiple sessions, usually five or more.

This trend is evident in studies involving youth with PPCS. When participants received repeated anodal stimulation over the left DLPFC, not only did their working memory and attentional performance improve, but these gains persisted several weeks beyond the final session. Such durability is rarely observed after a single exposure, pointing to the importance of cumulative stimulation in fostering neural change.

Adults with chronic mTBI show a similar pattern, although neuromodulatory responses in this stage may differ from those observed during the subacute recovery phase, when metabolic and inflammatory processes are still evolving. One study involving 10 sessions of 2 mA tDCS found significant improvements in sustained attention alongside increased prefrontal oxygenation—a physiological marker of enhanced neurovascular engagement (14). Notably, these effects extended beyond the stimulation period, further supporting the notion that repetition fosters not just short-term facilitation but longer-term functional restructuring.

Even outside mTBI, in aging populations with mild cognitive impairment, multi-session tDCS protocols have been shown to improve attention and executive function—especially when combined with task engagement (17). These improvements often emerge gradually, reinforcing the idea that plasticity is a process, not a quick fix.

Moreover, detailed task-based studies add another layer to this picture. One such experiment found that individuals receiving repeated tDCS sessions were better able to perform dual tasks with shorter psychological refractory periods—suggesting enhanced cognitive flexibility and processing speed under strain (12). These outcomes, taken together, emphasize that consistent, repeated stimulation is not simply preferable but may be essential for achieving therapeutic relevance in attentional recovery after mTBI.

### Exploring alternative cortical targets based on context

Although the left DLPFC is by far the most common target for tDCS in studies aiming to improve attention, it’s not necessarily the only one that matters—nor the best fit in every case. Some researchers have begun to question whether other brain regions might offer unique or complementary benefits, depending on the specific cognitive deficits or patient profile being addressed.

For example, in a crossover trial involving adults with persistent post-concussion symptoms, participants received tDCS targeting either the left DLPFC, the left temporal cortex, or a sham condition. As it is a study protocol, no outcome data are available; however, it highlights the hypothesis that stimulation site may differentially influence domain-specific cognitive functions such as executive processing and semantic memory (18). This kind of domain-specific response suggests that there’s more flexibility—and nuance—to montage selection than many earlier studies assumed.

Another compelling case comes from the world of athletics. A study in elite athletes experiencing mental fatigue compared the effects of stimulating three regions: DLPFC, the primary motor cortex (M1), and sham. Surprisingly, it was M1 stimulation—not the usual DLPFC target—that led to better performance on the Stroop task, particularly under fatigue. Athletes receiving M1 tDCS responded more accurately during cognitively taxing conditions (22). While it’s still unclear exactly why, one explanation is that M1 may modulate attentional performance through its tight links with sensorimotor control networks, especially in high-performance or stress-laden contexts.

What these studies underscore is that attention is not a one-size-fits-all construct—and neither should stimulation protocols be. The optimal target for tDCS may depend on the type of attention being rehabilitated (sustained vs. selective), the co-occurrence of fatigue or motor symptoms, or even the occupational demands of the individual. As the field matures, we may need to shift from fixed “best-practice” montages to more flexible, context-driven models that adjust stimulation sites based on both neuroanatomical rationale and patient-specific goals.

### Enhancing plasticity through task pairing and engagement

One of the most compelling findings in the neuromodulation literature is that tDCS does not work in isolation—it works better when the brain is actively engaged. This principle, often described through the lens of Hebbian plasticity (“cells that fire together wire together”), suggests that stimulating a brain region during task performance amplifies learning and functional recovery. In other words, the brain is more likely to change in useful ways if it’s being pushed and primed at the same time.

In studies involving individuals with moderate-to-severe TBI, pairing anodal tDCS over the left DLPFC with divided attention training led to clear improvements—not just in speed and accuracy but in how the brain functioned. Participants in these combined protocols showed reduced hyperactivation in frontal regions on fMRI after treatment, indicating a more efficient neural response to cognitive demand (23). These effects were not observed when either tDCS or training was applied alone.

This pattern is not unique to brain injury. In ADHD populations, multi-session tDCS paired with executive training has been associated with improved attentional control and reduced impulsivity, reinforcing the idea that brain stimulation is most effective when the circuits being targeted are actually in use (12). Similarly, in individuals with mild cognitive impairment, combining stimulation with memory or attention tasks yielded stronger and longer-lasting cognitive benefits compared to passive stimulation alone (21).

Why does this work? From a neurophysiological standpoint, task-based engagement increases regional blood flow, enhances cortical excitability, and may synchronize activity across networks like the frontoparietal attention system (20). One recent review of connectivity outcomes found that tDCS applied during cognitive tasks strengthened network coherence and improved information flow across attention-relevant regions (15). These kinds of network-level changes are difficult to achieve through training or stimulation on their own—but together, they seem to reinforce one another.

Ultimately, this body of work shifts the framing of tDCS: it’s not just a tool for “boosting” attention; it’s a way to amplify the brain’s response to cognitive effort. As such, future protocols should prioritize pairing stimulation with well-designed cognitive exercises that mirror the kinds of attention challenges patients face in daily life—whether it’s multitasking, resisting distractions, or maintaining focus during mentally draining activities.

### Methodological constraints and clinical implementation barriers

Despite the promising outcomes observed in preliminary trials, there are significant methodological and practical limitations that must be addressed before tDCS can be broadly adopted as a clinical intervention for attentional impairments in mTBI. First, most existing studies suffer from small sample sizes—often fewer than 30 participants per group—which reduces statistical power and generalizability. Meta-analyses are further hampered by heterogeneity in study protocols, including differences in electrode montage, stimulation intensity, session count, and outcome measures.

Second, many studies treat attention as a secondary outcome, focusing instead on global cognition or symptom burden. Furthermore, not all included studies directly measured attention as a primary outcome, with several relying on related cognitive domains such as working memory or executive function. While these domains overlap with attentional control, this distinction should be considered when interpreting the specificity of reported effects. This diminishes the interpretability of attentional findings and impedes targeted conclusions about tDCS efficacy in this domain. Additionally, most studies report only short-term outcomes, typically measured immediately post-treatment, without follow-up to assess durability or real-world transfer of benefits.

Additionally, while evidence from both Attention-Deficit/Hyperactivity Disorder and Mild Cognitive Impairment was incorporated to support cross-diagnostic interpretation, the available literature is more extensive for MCI than ADHD. As such, conclusions drawn from ADHD-related findings should be interpreted with caution, and further research is needed to strengthen evidence in this population.

Third, the clinical implementation of multi-session tDCS protocols presents logistical challenges. Repeated in-clinic visits may be impractical for many patients due to transportation, scheduling, or financial constraints. While home-based tDCS is emerging as a feasible alternative, especially in the context of expanding telehealth infrastructure, it raises concerns around safety, protocol adherence, and device regulation (11). One study highlighted that although remote-supervised tDCS is technically feasible, standardization in dosing, monitoring, and troubleshooting protocols is still lacking (14).

To overcome these barriers, future research must adopt more rigorous designs, implement standardized cognitive and neurophysiological outcome measures, and explore scalable delivery models that preserve treatment integrity without overburdening patients or healthcare systems.

### Recommendations for evidence-based advancement

Several crucial areas of research need to be addressed in order to convert the increasing promise of tDCS into clinical utility for attentional deficits following mTBI. First and foremost, randomized controlled trials must be sufficiently powered. A large portion of the literature currently in publication is based on small-sample pilot studies, which are useful for generating hypotheses but have limited generalizability. The evidence base would be significantly strengthened by adequately powered randomized controlled trials, standardized multi-session protocols (e.g., studies applying 10 sessions of 2 mA anodal tDCS (≈0.057–0.08 mA/cm^2^ depending on electrode size) over the left dorsolateral prefrontal cortex), and attention isolation as the primary endpoint.

Using validated outcome measures that are specific to attentional processes consistently is equally important. Attention-specific effects are obscured by the use of subjective symptom inventories or composite cognitive scores in many studies. The inclusion of performance-based tests—such as the Continuous Performance Test, Attention Network Test, or the Stroop Color-Word Test—would offer greater specificity. Patient-reported outcome measures that capture the functional impact of attentional difficulties in day-to-day life should be added to these.

Future research should use neurophysiological and neuroimaging biomarkers to gain a deeper understanding of the fundamental mechanisms underlying tDCS. Prior to and following intervention, methods like EEG, fNIRS, and fMRI can shed light on cortical excitability, regional blood flow, and functional connectivity. This could help identify which neural profiles predict a positive response to stimulation and enable researchers to ascertain whether improvements in cognition are correlated with changes in brain activity.

The problem of treatment durability must also be addressed by the field. Longer-term effects are unknown because the majority of studies to date evaluate results right after the last session. Determining whether observed improvements are temporary or suggest longer-lasting neural reorganization requires extended follow-up at three, six, and 12 months after treatment.

Furthermore, context-specific montage comparisons should be taken into consideration by researchers as evidence starts to point to the importance of alternative stimulation targets, such as the temporal cortex or primary motor cortex. For example, stimulation sites other than the conventional DLPFC may be beneficial for patients who present with motor fatigue or semantic memory impairments. Results from stratified protocols based on symptom profiles might be more reliable and customized.

Lastly, accessibility needs to be considered in implementation strategies. It can be logistically difficult to require patients to attend several in-clinic sessions, particularly for those who live in remote or underserved areas. Access could be significantly increased by remote, home-based tDCS systems that are supported by telehealth and clinically supervised. Even though preliminary feasibility studies have shown promise, more research is required to develop safe, standardized home delivery protocols that include monitoring and adherence safeguards.

Together, these suggestions offer a path forward for improving and expanding the application of tDCS in clinical neurorehabilitation. Standardized protocols, patient-centered delivery models, and more rigorous methodology can help the field move closer to establishing tDCS as an accessible, scientifically validated intervention for attentional recovery following mTBI.

### Final synthesis and future perspectives

Transcranial direct current stimulation has emerged as a promising and well-tolerated approach for addressing attentional deficits following mild traumatic brain injury. Multi-session anodal stimulation over the left dorsolateral prefrontal cortex has shown consistent benefits in improving sustained attention, enhancing response speed, and supporting executive function—especially when paired with targeted cognitive activities. These improvements are reflected not only in behavioral outcomes but also in neurophysiological changes, such as increased prefrontal activity and strengthened frontoparietal connectivity.

Despite encouraging findings, the current evidence base is still limited by small sample sizes, short-term follow-up, and inconsistent stimulation protocols. Questions remain around individual variability in treatment response, the durability of cognitive gains, and the best methods for delivering stimulation in routine care—including the role of home-based systems.

Moving forward, well-powered clinical trials with standardized designs and long-term outcome tracking will be essential. Integrating neurophysiological biomarkers may also improve protocol precision and help tailor interventions to individual needs. With continued refinement and thoughtful implementation, tDCS has the potential to become a practical and accessible tool in neurorehabilitation—offering patients a pathway to regain cognitive clarity, independence, and quality of life after concussion.

## Conclusion

Transcranial direct current stimulation is a promising treatment for attention problems after a mTBI, especially when given in multiple sessions over the dorsolateral prefrontal cortex. The evidence we have suggests that tDCS can improve cognitive control, increase sustained attention, and change how neurons work in networks related to attention, especially when used with tasks that require a lot of thinking. The first results are promising, but the literature is still limited because the sample sizes are small, the follow-up periods are short, and the methods are not always the same. Future research should focus on well-powered trials, standardized outcome measures that are specific to attentional domains, and the use of neurophysiological biomarkers. Also, personalized stimulation parameters and remote delivery models might help close the gap between how well something works in a lab and how well it works in the real world. tDCS could become a useful tool in neurorehabilitation for people recovering from mTBI if it keeps getting better.
